# Supplementary material for: Effects of Left Ventricular Unloading on Cardiac Function, Heart Failure Markers, and Autophagy in Rat Hearts with Acute Myocardial Infarction
Source: Int J Mol Sci. 2025 May 6;26(9):4422. doi: 10.3390/ijms26094422 (PMC12072880; doi:10.3390/ijms26094422)
Supplement: Supplementary file 1 [file ijms-26-04422-s001.zip › ijms-3597288-supplementary.pdf]

Table S1. Echocardiographic data before operation

groups	non-AMI	AMI	non-AMI + UL	AMI + UL	*p* values
2 days	(n = 4)	(n=4)	(n=4)	(n=4)	
BW, g	273 ± 3	275 ± 5	275 ± 3	278 ± 5	.85
HR, bpm	262 ± 3	281 ± 4	266 ± 14	270 ± 7	.43
LVEDD, mm	7.5 ± 0.1	7.4 ± 0.1	7.4 ± 0.1	7.4 ± 0.1	.71
LVESD, mm	4.2 ± 0.2	4.3 ± 0.2	4.1 ± 0.1	4.3 ± 0.1	.61
FS, %	43 ± 1	41 ± 2	45 ± 1	42 ± 1	.24
CO, ml/min	58 ± 3	53 ± 5	56 ± 5	52 ± 2	.67
SV, ml/beat	0.22 ± 0.01	0.19 ± 0.01	0.21 ± 0.01	0.19 ± 0.01	.30
14 days	(n = 6)	(n = 6)	(n = 5)	(n = 5)	
BW, g	278 ± 8	280 ± 3	274 ± 2	286 ± 9	.62
HR, bpm	260 ± 7	265 ± 3	276 ± 5	273 ± 5	.13
LVEDD, mm	7.3 ± 0.1	7.5 ± 0.1	7.3 ± 0.1	7.1 ± 0.4	.50
LVESD, mm	4.3 ± 0.1	4.2 ± 0.1	4.3 ± 0.2	3.8 ± 0.5	.40
FS, %	41 ± 1	42 ± 2	43 ± 1	48 ± 5	.26
CO, ml/min	56 ± 3	54 ± 5	53 ± 4	50 ± 2	.75
SV, ml/beat	0.22 ± 0.01	0.20 ± 0.02	0.19 ± 0.02	0.18 ± 0.01	.50

Data are expressed as mean ± SEM. AMI, acute myocardial infarction; BW, body weight; CO, cardiac output; FS, fractional shortening; HR, heart rate; LVEDD, LV end-diastolic dimension; LVESD, LV end-systolic dimension; SV, stroke volume; UL, unloading. Data were analyzed using one-way ANOVA to detect differences.

Table S2. Echocardiographic data before euthanization

groups	non-AMI	AMI	non-AMI + UL	AMI + UL	F values for two-way ANOVA: main effect AMI	main effect UL	interaction
2 days	(n = 4)	(n=4)	(n=4)	(n=4)			
BW, g	263 ± 3	255 ± 6	250 ± 0.0	253 ± 5	0.35	3.18	1.41
HR, bpm	250 ± 7	210 ± 32	218 ± 12	237 ± 12	0.35	0.015	2.51
LVEDD, mm	7.5 ± 0.2	7.3 ± 0.2	4.1 ± 0.4	5.1 ± 0.4	1.29	70.1****	3.37
LVESD, mm	4.5 ± 0.2	4.3 ± 0.2	3.5 ± 0.3	4.9 ± 0.5	5.24*	1.89	1.7
FS, %	40 ± 1	33 ± 5	13 ± 1	5.7 ± 1	6.4*	96.4****	0.007
14 days	(n = 6)	(n = 6)	(n = 5)	(n = 5)			
BW, g	291 ± 5	298 ± 5	310 ± 6	306 ± 9	0.045	4.27	0.72
HR, bpm	245 ± 8	255 ± 5	230 ± 7	255 ± 6	6.74*	1.31	1.12
LVEDD, mm	7.4 ± 0.1	9.0 ± 0.3	4.5± 0.4	5.0± 0.5	9.14**	98.2****	2.12
LVESD, mm	2.5± 0.1	2.4 ± 0.2	2.1 ± 0.2	2.0 ± 0.3	0.34	3.86	0.0017
FS, %	40 ± 1	27 ± 2	25 ± 4	15 ± 3	24***	35.5****	0.33

Data are expressed as mean ± SEM. AMI, acute myocardial infarction; BW, body weight; FS, fractional shortening; HR, heart rate; LVEDD, LV end-diastolic dimension; LVESD, LV end-systolic dimension; UL, unloading. *p < .05, **p < .01, ***p < .001, and ****p < .0001.
